# Efficacy and safety of rotigotine in elderly patients with Parkinson’s disease in comparison with the non-elderly: a post hoc analysis of randomized, double-blind, placebo-controlled trials

**DOI:** 10.1007/s00415-017-8671-0

**Published:** 2017-11-21

**Authors:** Masahiro Nomoto, Hirotaka Iwaki, Hiroyuki Kondo, Masaya Sakurai

**Affiliations:** 1 Department of Neurology and Clinical Pharmacology, Ehime University Hospital, Shitsukawa, Toon, Ehime Japan; 2 Department of Neurology and Clinical Pharmacology, Ehime University Graduate School of Medicine, Shitsukawa, Toon, Ehime Japan; 3 Department of Medical Affairs, Otsuka Pharmaceutical Co., Ltd, Tokyo, Japan

**Keywords:** Rotigotine, Post hoc analysis, Elderly patients, Parkinson’s disease, Efficacy, Safety

## Abstract

Rotigotine—a non-ergot dopamine agonist—has two advantages; it can stimulate all dopamine receptors (D1–D5) like innate dopamine, and its transdermal administration provides continuous dopaminergic stimulation. The age of the patient impacts the effect and adverse events of anti-parkinsonian treatment. We conducted a post hoc analysis on three randomized, double-blind, placebo-controlled trials performed in Japan to clarify the difference of anti-parkinsonian treatment in elderly and non-elderly patients. Data from two combination therapy trials (with levodopa) in advanced stage Parkinson’s disease patients and one monotherapy trial in early stage patients were pooled and grouped by age (non-elderly aged < 70, elderly aged 70 +). In each age group, efficacy of rotigotine was compared to placebo. In the combination therapy, total Unified Parkinson’s Disease Rating Scale Part III scores and some subtotal scores, including those for tremor, akinesia and gait disturbance, significantly improved in both elderly and non-elderly patients. Regarding safety, the incidence of total adverse event tended to be lower in elderly patients than non-elderly patients, although it was not significant. No difference was observed in maintenance dosage of rotigotine between the two groups. In conclusion, the improvement in motor symptoms and frequency of adverse events were shown to be similar in elderly and non-elderly patients with rotigotine–levodopa combination therapy. Further, there was no major difference in maintenance dosage of rotigotine between the age groups. These results suggest good tolerability of rotigotine among elderly patients.

**Electronic supplementary material:**

The online version of this article (10.1007/s00415-017-8671-0) contains supplementary material, which is available to authorized users.

## Introduction

The Parkinson’s disease (PD) patient population has become older along with Japanese population as a whole. Among all PD patients, approximately 80% were 70 years of age or older, according to a national survey in 2014 [1].

Levodopa is recommended as an initial treatment for naive elderly Japanese PD patients due to its efficacy and safety [2]. However, motor complications are more common for levodopa treatment than for dopamine agonists [2]. Reconsideration of the dosage and the treatment pattern is needed if motor complications occur. Especially for off symptoms, one option is to administer additional PD drugs other than levodopa, such as dopamine agonists [2]. However, with use of either levodopa or a dopamine agonist, it becomes difficult to increase the dosage and to manage motor symptoms when psychological symptoms are found [3]. The dilemma for elderly PD patients is that the dosage of dopaminergic agents cannot be increased; consequently, motor symptoms cannot be controlled, compromising activities of daily living (ADL) [3]. Inability to perform ADL is a serious problem not only for the patients themselves, but also for their family members and caregivers [4].

A PD treatment based on the continuous dopaminergic stimulation theory [5–7] has been expected. Furthermore, since dysphagia often occurs due to neurodegeneration, and taking many oral medications can be a big burden for such a patient, a pharmaceutical formulation which is unaffected by swallowing or intestinal absorption is important. Thus, the rotigotine—a non-ergot dopamine agonist—transdermal patch was developed to meet this need [8, 9]. Rotigotine stimulates all dopamine receptors (D1–D5), and a compatibility profile similar to dopamine [10] has been reported. If motor symptoms can be improved by additional administration of rotigotine without adverse events (AEs), a decline in ADL can be prevented thereby benefiting patients, their family members, and caregivers [4].

There are several reports on the use of rotigotine among elderly patients [11–13]. However, there are no reports regarding the efficacy and safety of rotigotine comparing PD patients under and over age 70, which is the reference age for categorization as elderly when prescribing dopamine agonists in the Japanese guideline [2], the Treatment Guidelines published by *Neurology* [14], and the Canadian Guideline [15]. Therefore, we performed a post hoc analysis on the results of three clinical trials conducted in Japan [16–18] to examine the efficacy and safety of rotigotine treatment for elderly and non-elderly PD patients separately.

## Methods

### Study design and patients

We carried out a post hoc analysis of all randomized, double-blind, placebo-controlled, and parallel-group comparison trials (Phase II or III) that had been conducted in Japan to evaluate the efficacy and safety of rotigotine in elderly PD patients (defined as age 70 and older) compared to non-elderly PD patients (defined as under age 70). The study designs included two types of therapies: combination therapy with levodopa in advanced stage patients (identifier: NCT01628848, NCT01628926) [16, 17] and monotherapy in early stage patients (identifier: NCT01628965) [18]. Primary endpoints were change in total Unified Parkinson’s Disease Rating Scale (UPDRS) Part III score from baseline to the end of treatment for the trials of combination therapy, and sum of total UPDRS Part II and Part III scores for monotherapy. Data from the combination therapy trials were pooled and grouped by age. The dosage of levodopa did not change during the study period. In all three trials, PD patients (30–79 years old) were given rotigotine transdermal patches delivering 2–16 mg/24 h of rotigotine for 12 or 16 weeks. The patients with placebo treatment were given the same sized patches as the rotigotine treatment (2–16 mg/24 h). The parallel “dosage” of placebo was defined by the size of the patch.

### Outcome measures

The treatment efficacy of rotigotine was compared to placebo in each age group, and safety was compared between age groups.

Efficacy was examined as the absolute change from baseline to the end of a 12-week of titration/maintenance period in each of the following measures: total UPDRS Part III score, the sum of UPDRS Part II and Part III scores (only in monotherapy), the sum of related UPDRS items for each motor symptom [tremor (items 16, 20, 21), rigidity (item 22), akinesia (items 8, 10–12, 18, 19, 23–26, 31), and postural instability (items 27, 28, 30)], gait disturbance (items 13–15, 29), each UPDRS item score related to motion and ADLs (items 3, 4, 10–14, 27, 29, 30), mood (total UPDRS Part I score), and OFF-time (only in combination therapy). Safety was assessed based on AEs; those often reported for dopamine agonists were defined as especially remarkable AEs (sudden onset of sleep, hallucinations including visual and auditory hallucinations, delusion, nausea, vomiting, orthostatic hypotension, and somnolence).

### Statistics

Efficacy and safety were examined, respectively, using the full analysis set (randomized patients taking treatment at least once, and post-baseline efficacy evaluation at least once; FAS) and the safety set (all randomized patients taking treatment at least once; SS). For efficacy, primary statistical inference was based on a *t* test for imputed data with the last observation carried forward. A mixed-effect model repeated measure (MMRM) with the baseline values as a covariate, and group, time point, and interaction of group and time point as factors was also performed as a sensitivity analysis, especially for the total UPDRS Part III score. For safety, the cumulative incidence of each AE was summarized and the difference between age groups was assessed by a Chi-square test. Statistical significance was assessed as *P* < 0.05 for both efficacy and safety. Excel 2013 (Microsoft, Redmond, WA, USA), JMP Version 11.0 or above and SAS Version 9.3 or above (SAS institute, Cary, NY, USA) were used.

## Results

### Baseline characteristics and dosage of drugs

The numbers of pooled patients in the combination therapy groups were 255 in the rotigotine group (levodopa–rotigotine group) and 172 in the placebo group (levodopa–placebo group) in the SS, and 250 and 170, respectively, in the FAS. The numbers of patients in the monotherapy groups were 90 in the rotigotine group and 90 in the placebo group in the SS, and 88 and 88 in the FAS, respectively (supplementary material: Table S1). The mean age of each group was 73.2–74.2 for elderly and 61.3–61.8 for non-elderly (Table 1A). Baseline characteristics including age, disease duration, and Hoehn and Yahr (HY) staging scale were not different between the rotigotine treatment group and placebo group for the same age group and therapy type (monotherapy or combination therapy) (Table 1A). The results were not different from those in the SS (Table 1B).

**Table 1:** Baseline characteristics in (A) full analysis set and (B) safety set in (a) combination therapy with levodopa and (b) monotherapy

(A)													
	(a) Combination therapy with levodopa												
	Elderly					Non-elderly							
	Rotigotine		Placebo			Rotigotine				Placebo			
	*n*		*n*			*n*				*n*			
Age (years), mean ± SD	86	73.6 ± 2.8	68	73.2 ± 2.7		164		61.3 ± 6.9		102		61.4 ± 7.0	
Weight (kg), mean ± SD	86	52.7 ± 11.4	68	57.5 ± 10.6		164		56.1 ± 10.7		102		57.4 ± 9.4	
Duration of PD (years), mean ± SD	86	7.4 ± 6.4	68	6.2 ± 3.5		164		7.0 ± 4.6		102		6.1 ± 3.9	
Levodopa dose at first intervention (mg), mean ± SD	86	370.9 ± 180.9	68	342.6 ± 125.6		164		356.1 ± 145.5		102		354.2 ± 150.5	
Sex (male), number (%)	86	33 (38.4)	68	40 (58.8)		164		62 (37.8)		102		46 (45.1)	
HY scale, number (%)													
0	–	–	–	–		–		–		–		–	
1	–	–	–	–		–		–		–		–	
1.5	–	–	–	–		–		–		–		–	
2	86	16 (18.6)	68	15 (22.1)		164		40 (24.4)		102		22 (21.6)	
2.5	86	14 (16.3)	68	12 (17.6)		164		45 (27.4)		102		25 (24.5)	
3	86	47 (54.7)	68	33 (48.5)		164		65 (39.6)		102		48 (47.1)	
4	86	9 (10.5)	68	8 (11.8)		164		14 (8.5)		102		7 (6.9)	
5	–	–	–	–		–		–		–		–	
UPDRS score, mean ± SD													
Part I	86	1.07 ± 1.34	68		1.18 ± 1.54		164		0.92 ± 1.46		102		0.71 ± 1.03
Part II (on)	86	9.40 ± 5.59	68		9.24 ± 6.07		164		8.24 ± 5.84		102		7.20 ± 5.48
Part II (off)	49	14.51 ± 8.36	42		16.55 ± 9.12		116		16.00 ± 8.91		75		13.97 ± 6.82
Part II (average)	86	11.31 ± 5.78	68		11.57 ± 6.68		163		11.26 ± 6.36		102		10.12 ± 63.62
Part III	86	27.59 ± 11.38	68		26.91 ± 10.46		164		26.10 ± 11.09		102		25.30 ± 10.29
Part II + III	86	2.87 ± 2.76	67		2.58 ± 2.35		164		3.48 ± 2.81		102		3.34 ± 2.39

	(b) Monotherapy							
	Elderly				Non-elderly			
	Rotigotine		Placebo		Rotigotine		Placebo	
	*n*		*n*		*n*		*n*	
Age (years), mean ± SD	28	73.3 ± 2.6	31	74.2 ± 3.0	60	61.3 ± 6.9	57	61.8 ± 6.0
Weight (kg), mean ± SD	28	55.2 ± 10.9	31	54.0 ± 8.7	60	56.8 ± 9.2	57	55.4 ± 9.5
Duration of PD (years), mean ± SD	28	1.3 ± 1.3	31	1.5 ± 1.7	60	2.3 ± 1.9	57	2.0 ± 2.0
Sex (male), number (%)	28	12 (42.9)	31	14 (45.2)	60	21 (35.0)	57	23 (40.4)
HY scale, number (%)								
0	–	–	–	–	–	–	–	–
1	28	2 (7.1)	31	0 (0.0)	60	10 (16.7)	57	5 (8.8)
1.5	28	3 (10.7)	31	5 (16.1)	60	5 (8.3)	57	7 (12.3)
2	28	11 (39.3)	31	14 (45.2)	60	23 (38.3)	57	22 (38.6)
2.5	28	5 (17.9)	31	5 (16.1)	60	6 (10.0)	57	7 (12.3)
3	28	7 (25.0)	31	7 (22.6)	60	16 (26.7)	57	16 (28.1)
4	–	–	–	–	–	–	–	–
5	–	–	–	–	–	–	–	–
UPDRS score, mean ± SD								
Part I	28	1.00 ± 1.66	31	0.81 ± 1.01	60	0.67 ± 1.05	57	0.44 ± 0.78
Part II	28	6.86 ± 4.72	31	8.03 ± 3.89	60	6.80 ± 3.56	57	7.12 ± 3.56
Part III	28	20.89 ± 9.06	31	20.90 ± 9.16	60	19.82 ± 9.36	57	20.74 ± 9.77
Part II + III	28	0.25 ± 0.44	31	0.23 ± 0.43	60	0.32 ± 0.54	57	0.18 ± 0.38

(B)								
	(a) Combination therapy with levodopa							
	Elderly				Non-elderly			
	Rotigotine		Placebo		Rotigotine		Placebo	
	*n*		*n*		*n*		*n*	
Age (years), mean ± SD	87	73.6 ± 2.8	69	73.2 ± 2.6	168	61.4 ± 6.9	103	61.4 ± 7.0
Weight (kg), mean ± SD	87	52.8 ± 11.4	69	57.3 ± 10.7	168	56.0 ± 10.6	103	57.5 ± 9.6
Duration of PD (years), mean ± SD	87	7.3 ± 6.3	69	6.1 ± 3.5	168	6.9 ± 4.6	103	6.1 ± 3.9
Levodopa dose at first intervention (mg), mean ± SD	87	370.1 ± 180.0	69	342.0 ± 124.7	168	365.4 ± 196.5	103	353.6 ± 149.8
Sex (male), number (%)	87	34 (39.1)	69	40 (58.0)	168	62 (36.9)	103	47 (45.6)
HY scale, number (%)								
0	–	–	–	–	–	–	–	–
1	–	–	–	–	–	–	–	–
1.5	–	–	–	–	–	–	–	–
2	87	16 (18.4)	69	16 (23.2)	168	41 (24.4)	103	22 (21.4)
2.5	87	14 (16.1)	69	12 (17.4)	168	45 (26.8)	103	25 (24.3)
3	87	48 (55.2)	69	33 (47.8)	168	66 (39.3)	103	49 (47.6)
4	87	9 (10.3)	69	8 (11.6)	168	16 (9.5)	103	7 (6.8)
5	–	–	–	–	–	–	–	–
UPDRS score, mean ± SD								
Part I	87	1.07 ± 1.33	69	1.19 ± 1.34	168	0.96 ± 1.49	103	0.70 ± 1.03
Part II (on)	87	9.41 ± 5.56	69	9.17 ± 6.05	168	8.33 ± 5.91	103	7.18 ± 5.45
Part II (off)	50	14.62 ± 8.31	43	16.40 ± 9.07	118	15.99 ± 8.84	76	13.95 ± 6.78
Part II (average)	87	11.36 ± 5.76	69	11.51 ± 6.65	167	11.31 ± 6.35	103	10.11 ± 5.30
Part III	87	27.77 ± 11.43	69	26.68 ± 10.56	168	26.47 ± 11.57	103	25.45 ± 10.34
Part II + III	87	2.90 ± 2.76	68	2.60 ± 2.34	168	3.46 ± 2.81	103	3.35 ± 2.38

	(b) Monotherapy							
	Elderly				Non-elderly			
	Rotigotine		Placebo		Rotigotine		Placebo	
	*n*		*n*		*n*		*n*	
Age (years), mean ± SD	30	73.3 ± 2.5	31	74.2 ± 3.0	60	61.3 ± 6.9	59	61.6 ± 6.4
Weight (kg), mean ± SD	30	55.0 ± 10.5	31	54.0 ± 8.7	60	56.8 ± 9.2	59	55.4 ± 9.6
Duration of PD (years), mean ± SD	30	1.3 ± 1.3	31	1.5 ± 1.7	60	2.3 ± 1.9	59	2.1 ± 2.0
Sex (male), number (%)	30	13 (43.3)	31	14 (45.2)	60	21 (35.0)	59	25 (42.4)
HY scale, number (%)								
0	–	–	–	–	–	–	–	–
1	30	2 (6.7)	31	0 (0.0)	60	10 (16.7)	59	5 (8.5)
1.5	30	3 (10.0)	31	5 (16.1)	60	5 (8.3)	59	7 (11.9)
2	30	13 (43.3)	31	14 (45.2)	60	23 (38.3)	59	23 (39.0)
2.5	30	5 (16.7)	31	5 (16.1)	60	6 (10.0)	59	7 (11.9)
3	30	7 (23.3)	31	7 (22.6)	60	16 (26.7)	59	17 (28.8)
4	–	–	–	–	–	–	–	–
5	–	–	–	–	–	–	–	–
UPDRS score								
Part I	30	1.03 ± 1.61	31	0.81 ± 1.01	60	0.67 ± 1.05	59	0.42 ± 0.77
Part II	30	6.97 ± 4.57	31	8.03 ± 3.89	60	6.80 ± 3.56	59	7.41 ± 4.64
Part III	30	20.73 ± 8.78	31	20.90 ± 9.16	60	19.82 ± 9.36	59	21.36 ± 10.89
Part II + III	30	0.30 ± 0.53	31	0.23 ± 0.43	60	0.32 ± 0.54	59	0.17 ± 0.38

*SD* standard deviation, *HY scale* modified Hoehn and Yahr scale, *UPDRS score* Unified Parkinson’s Disease Rating Scale score

No differences were observed in the mean dosages of levodopa at baseline for combination therapy between the levodopa–rotigotine and levodopa–placebo groups, nor between elderly and non-elderly patients (Table 1A). The mean (standard deviation, SD) maintenance dosages of rotigotine for the levodopa–rotigotine combination therapy group and rotigotine monotherapy group were 13.4 (3.8) mg/24 h and 13.1 (3.9) mg/24 h for elderly patients and 12.5 (3.9) mg/24 h and 12.7 (4.0) mg/24 h for non-elderly patients, respectively (supplementary material: Table S2).

### Efficacy and safety in combination therapy in advanced stage PD patients

**Fig. 1:**
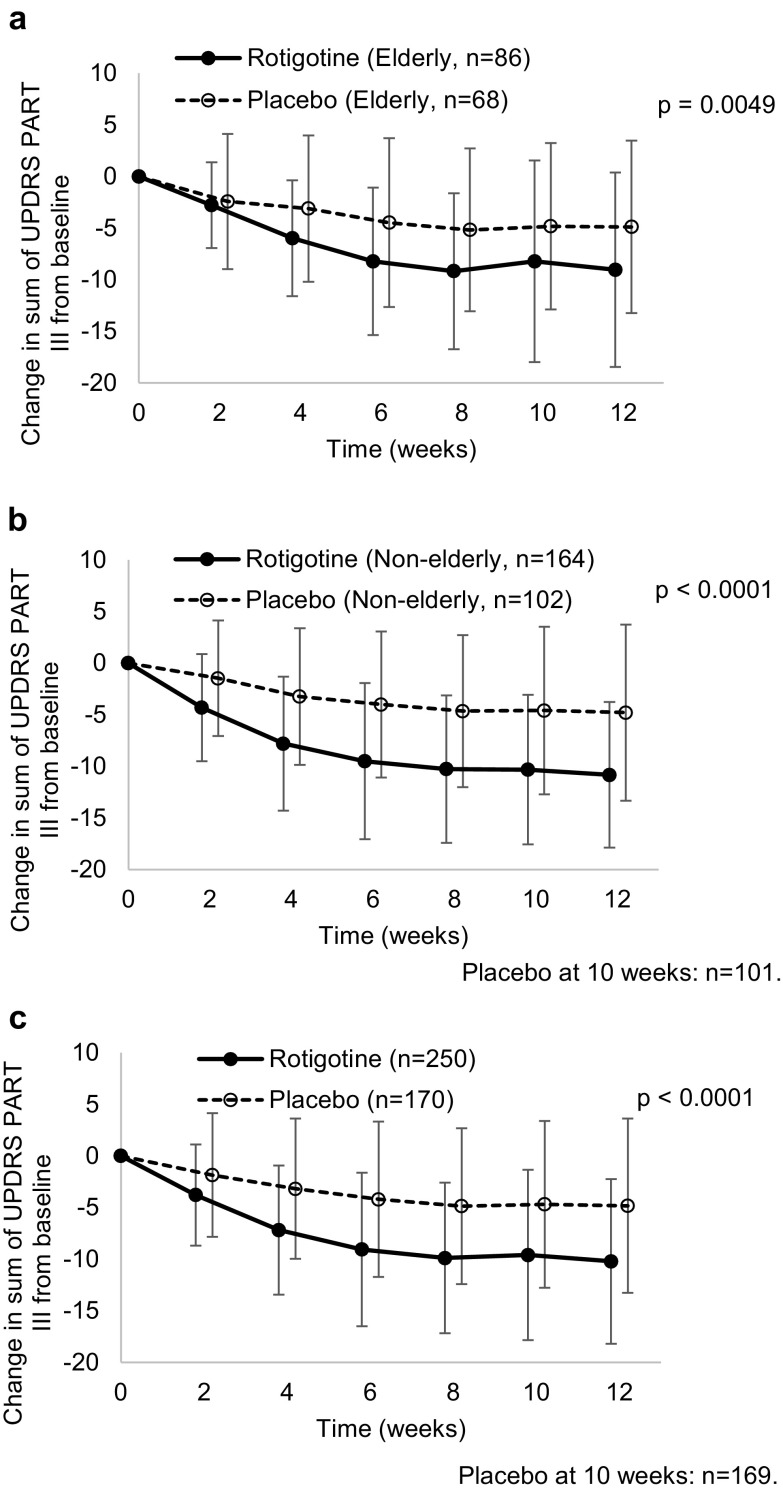
Change in total UPDRS Part III score from baseline for 12 weeks with combination therapy in the **a** elderly, **b** non-elderly and **c** total patient groups; black and white circles show the mean value of the change in the score from baseline during treatment with rotigotine and placebo, respectively; error bars show standard deviation; *P* value is in the 12th week

Efficacy in the levodopa–rotigotine group was compared to that in the levodopa–placebo group. The total UPDRS Part III score in both elderly and non-elderly patients decreased in both the levodopa–rotigotine and levodopa–placebo groups (Fig. 1). The magnitude of decline in the levodopa–rotigotine group was greater than the levodopa–placebo group in both elderly and non-elderly patients (*P* = 0.0049 for elderly, and *P* < 0.0001 for non-elderly). No difference was seen by MMRM (not reported). Table S3A (supplementary material) shows the change in UPDRS scores related to motor symptoms. Greater decreases were seen with levodopa–rotigotine treatment than with placebo for non-elderly patients (*P* = 0.0006, *P* < 0.0001, *P* < 0.0001, and *P* < 0.0001 for tremor, rigidity, akinesia, and postural instability, respectively). Greater improvements were also seen in elderly patients (*P* = 0.0020 and *P* = 0.0147 for tremor and akinesia, respectively). The scores for gait disturbance showed greater improvement in both elderly and non-elderly patients (*P* = 0.0050 for elderly, and *P* = 0.0007 for non-elderly) (supplementary material: Table S3B).

**Fig. 2:**
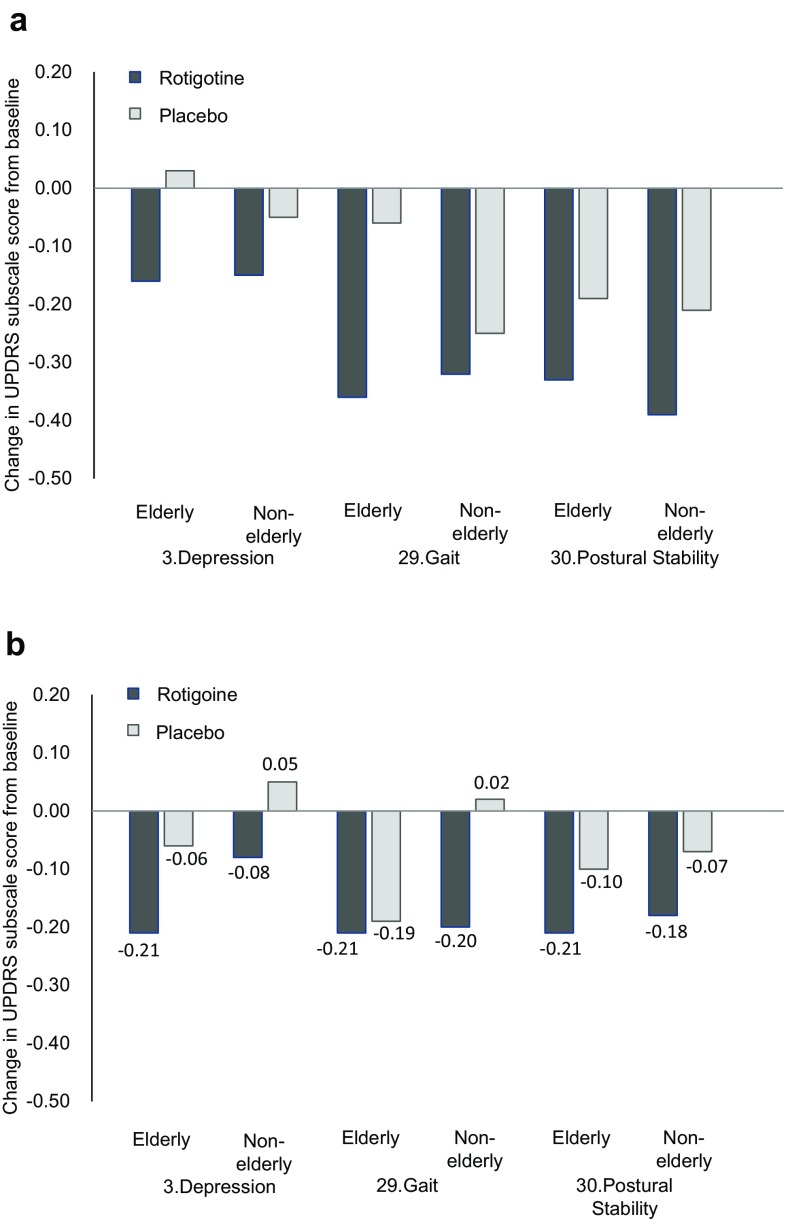
Change in UPDRS item scores for depression, gait, and postural stability from baseline to the end of maintenance period with **a** combination therapy with levodopa, and **b** monotherapy

Among the UPDRS items related to motion and ADLs, mean scores decreased with levodopa–rotigotine treatment in all items, and the changes were greater than levodopa–placebo treatment for some scores (Fig. 2a and supplementary material: Table S4). Motivation/initiative (item 4) showed greater improvement in both elderly and non-elderly patients (*P* = 0.0207 for elderly, and *P* = 0.0048 for non-elderly). Other items that showed greater improvement in elderly patients were depression (item 3), hygiene (item 11), turning in bed and adjusting bed clothes (item 12), and gait (item 29) (*P* = 0.0286, *P* = 0.0327, *P* = 0.0250, and *P* = 0.0035, respectively). Other items that showed greater improvement in non-elderly patients were falling (item 13), freezing when walking (item 14), arising from chair (item 27), and postural stability (item 30) (*P* = 0.0446, *P* = 0.0036, *P* = 0.0016, and *P* = 0.0216, respectively).

There was greater improvement in OFF-time among non-elderly patients than levodopa-placebo treatment (*P* = 0.0005), although this measure did not improve significantly for elderly patients (supplementary material: Table S5).

The combined patient population experienced a reduced total UPDRS Part I score (*P* = 0.0133), but the magnitude of the decreases was not significant for the age groups separately (supplementary material: Table S6).

In terms of safety, the cumulative incidences of AEs were compared between elderly and non-elderly patients. The cumulative incidences of remarkable AEs, and those occurring in at least 5% of study patients with rotigotine treatment, and those with placebo treatment as a reference, are shown in Table 2A, B, respectively. The total cumulative incidence in elderly patients was not higher than that in non-elderly patients. Application site reactions were less frequent in elderly patients compared to non-elderly patients (*P* = 0.0165). In terms of remarkable AEs, somnolence, vomiting, and orthostatic hypotension occurred less frequently in elderly patients than in non-elderly patients, although the difference was not statistically significant. The cumulative incidences of visual hallucination and hallucination (not including auditory hallucination) tended to be higher in elderly patients than those in non-elderly patients.

**Table 2:** Cumulative incidences of adverse events for (A) rotigotine treatment and (B) placebo: those occurring ≥ 5%, and those defined as remarkable adverse events

(A)										
Adverse event (PT)	Combination therapy (rotigotine with levodopa)					Monotherapy (levodopa)				
	Elderly (*n* = 87)		Non-elderly (*n* = 168)		*p* value^†^	Elderly (*n* = 30)		Non-elderly (*n* = 60)		*p* value^†^
	*n*	Ratio (%)	*n*	Ratio (%)		*n*	Ratio (%)	*n*	Ratio (%)	
Total	76	87.4	155	92.3	0.2034	24	80.0	54	90.0	0.1883
Application site reactions	37	42.5	98	58.3	0.0165	10	33.3	32	53.3	0.0730
Nasopharyngitis	17	19.5	29	17.3	0.6537	4	13.3	7	11.7	0.8200
Nausea^a^	15	17.2	27	16.1	0.8113	2	6.7	19	31.7	0.0082
Dyskinesia	9	10.3	30	17.9	0.1141	0	0.0	1	1.7	0.4770
Somnolence^a^	6	6.9	17	10.1	0.3944	6	20.0	7	11.7	0.2891
Visual hallucination^a^	8	9.2	13	7.7	0.6882	1	3.3	2	3.3	1.0000
Vomiting^a^	6	6.9	14	8.3	0.6858	2	6.7	12	20.0	0.0999
Contusion	7	8.0	6	3.6	0.1235	1	3.3	0	0.0	0.1550
Loss of appetite	5	5.7	7	4.2	0.5721	2	6.7	3	5.0	0.7449
Blood creatinine phosphokinase increase	3	3.4	9	5.4	0.4950	1	3.3	3	5.0	0.7176
Application site pruritus	5	5.7	6	3.6	0.4175	0	0.0	0	0.0	–
Dizziness	7	8.0	4	2.4	0.0348	0	0.0	0	0.0	–
Fall	5	5.7	5	3.0	0.2798	0	0.0	0	0.0	–
Orthostatic hypotension^a^	1	1.1	6	3.6	0.2618	0	0.0	0	0.0	–
Hallucination^a^	4	4.6	2	1.2	0.0888	4	13.3	0	0.0	0.0038
Auditory hallucination^a^	0	0.0	3	1.8	0.2099	0	0.0	0	0.0	–
Delusion^a^	1	1.1	2	1.2	0.9770	0	0.0	0	0.0	–
Sudden onset of sleep^a^	1	1.1	0	0.0	0.1638	1	3.3	0	0.0	0.1550
Constipation	3	3.4	8	4.8	0.6245	3	10.0	9	15.0	0.5107
Insomnia	0	0.0	6	3.6	0.0745	2	6.7	4	6.7	1.0000
Back pain	0	0.0	6	3.6	0.0745	2	6.7	1	1.7	0.2129
Diarrhea	2	2.3	2	1.2	0.4995	0	0.0	3	5.0	0.2129
Weight loss	1	1.1	2	1.2	0.9770	2	6.7	0	0.0	0.0431
Peripheral edema	1	1.1	1	0.6	0.6343	2	6.7	0	0.0	0.0431
Hypokalemia	0	0.0	0	0.0	–	2	6.7	0	0.0	0.0431

(B)								
Adverse event (PT)	Combination therapy (levodopa with placebo)				Monotherapy (placebo)			
	Elderly (*n* = 69)		Non-elderly (*n* = 103)		Elderly (*n* = 31)		Non-elderly (*n* = 59)	
	*n*	Ratio (%)	*n*	Ratio (%)	*n*	Ratio (%)	*n*	Ratio (%)
Total	53	76.8	83	80.6	19	61.3	46	78.0
Application site reactions	9	13.0	17	16.5	6	19.4	14	23.7
Nasopharyngitis	11	15.9	15	14.6	4	12.9	11	18.6
Nausea^a^	4	5.8	8	7.8	0	0.0	5	8.5
Dyskinesia	3	4.3	5	4.9	0	0.0	0	0.0
Somnolence^a^	1	1.4	2	1.9	1	3.2	3	5.1
Visual hallucination^a^	2	2.9	3	2.9	0	0.0	0	0.0
Vomiting^a^	1	1.4	2	1.9	1	3.2	0	0.0
Contusion	2	2.9	7	6.8	3	9.7	4	6.8
Loss of appetite	1	1.4	2	1.9	0	0.0	0	0.0
Blood creatinine phosphokinase increase	1	1.4	3	2.9	1	3.2	1	1.7
Application site pruritus	2	2.9	2	1.9	0	0.0	0	0.0
Dizziness	1	1.4	2	1.9	2	6.5	2	3.4
Fall	2	2.9	6	5.8	1	3.2	1	1.7
Orthostatic hypotension^a^	4	5.8	2	1.9	0	0.0	1	1.7
Hallucination^a^	2	2.9	1	1.0	0	0.0	0	0.0
Auditory hallucination^a^	0	0.0	1	1.0	0	0.0	0	0.0
Delusion^a^	0	0.0	0	0.0	0	0.0	0	0.0
Sudden onset of sleep^a^	0	0.0	0	0.0	0	0.0	0	0.0
Constipation	2	2.9	1	1.0	1	3.2	4	6.8
Insomnia	2	2.9	2	1.9	0	0.0	2	3.4
Back pain	2	2.9	4	3.9	0	0.0	1	1.7
Diarrhea	1	1.4	2	1.9	2	6.5	1	1.7
Weight loss	0	0.0	0	0.0	0	0.0	0	0.0
Peripheral edema	1	1.4	3	2.9	0	0.0	0	0.0
Hypokalemia	0	0.0	0	0.0	0	0.0	0	0.0

^a^Symptom defined as a remarkable adverse event

^†^For comparison between elderly and non-elderly groups

### Efficacy and safety in monotherapy in early stage PD patients

**Fig. 3:**
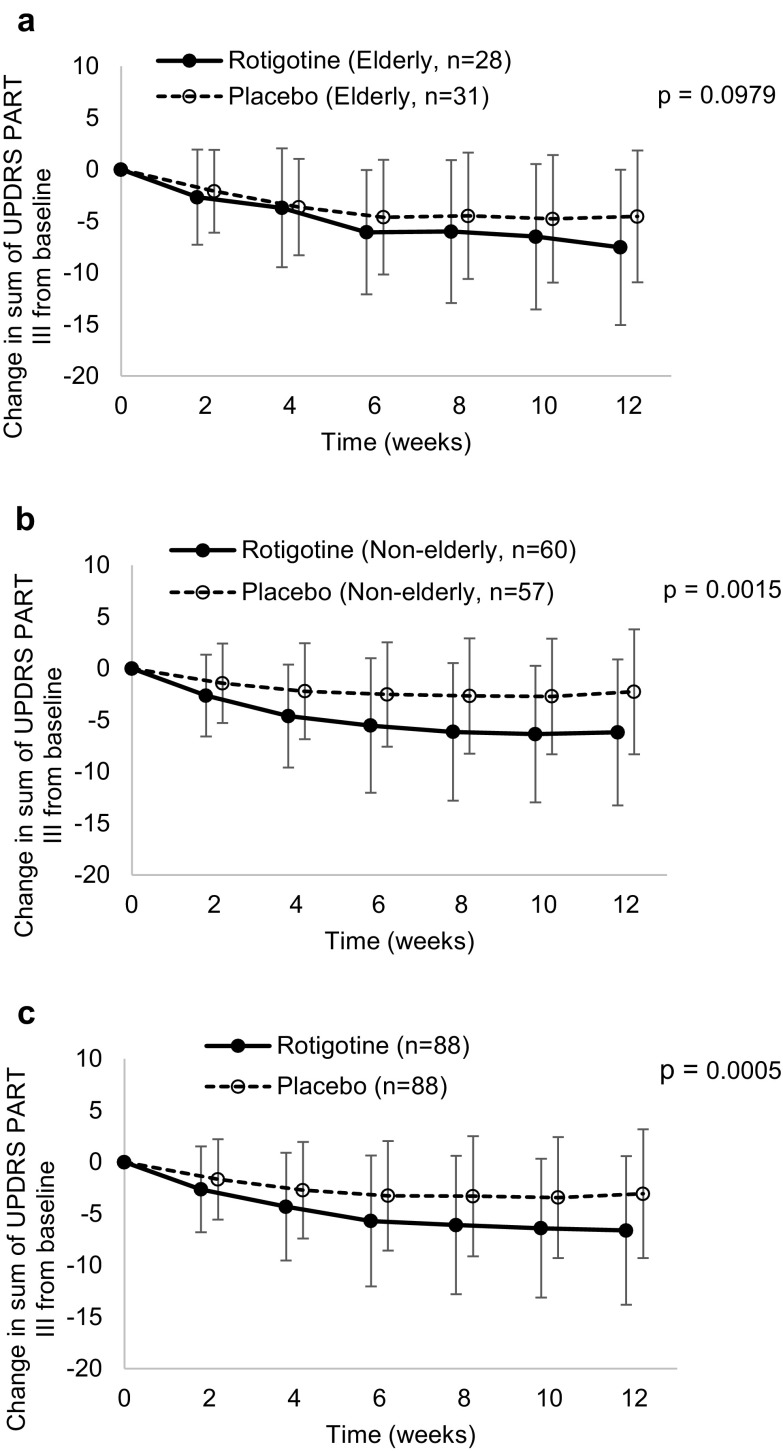
Change in total UPDRS Part III score from baseline for 12 weeks with monotherapy in the **a** elderly, **b** non-elderly and **c** total patient groups; black and white circles show the mean value of the change in the score from baseline during treatment with rotigotine and placebo, respectively; error bars show standard deviation; *P* value is in the 12th week

Efficacy in the rotigotine group was compared with that in the placebo group as well. The total UPDRS Part III score and the sum of UPDRS Part II and Part III scores decreased in both elderly and non-elderly patients in the rotigotine group compared to the placebo group (Fig. 3 and supplementary material: Table S7). The differences were not significant for elderly patients.

The scores related to motor symptoms (tremor and akinesia) and gait disturbance improved with rotigotine treatment compared to placebo only in non-elderly patients (*P* = 0.0015, *P* = 0.0033, and *P* = 0.0273 for tremor, akinesia and gait disturbance, respectively) (supplementary material: Table S3A and S3B). The scores on other items including freezing when walking (item 14) and gait (item 29) also showed greater improvement in non-elderly patients (*P* = 0.0408 and *P* = 0.0244, respectively) compared to placebo, but this comparison was not significant in elderly patients (Fig. 2b and supplementary material: Table S4).

The total UPDRS Part I score decreased more in the rotigotine group compared to the placebo group, but the differences were not significant in either age group (supplementary material: Table S6).

The cumulative incidence of AEs was compared between elderly and non-elderly monotherapy patients as well. The total cumulative incidence of AEs tended to be less frequent in elderly compared to non-elderly patients (Table 2). The occurrences of application site reactions, vomiting, and nausea were lower with significant or close to significant difference (*P* = 0.0730, 0.0999, and 0.0082, respectively) in elderly patients than non-elderly patients. No incident of orthostatic hypotension was seen in either age group. Hallucination (not including visual hallucination auditory hallucination) occurred only in elderly patients.

Overall, the safety profile showed the same trend in both age groups in monotherapy as well as combination therapy.

## Discussion

For combination therapy of rotigotine with levodopa in advanced stage patients, the total UPDRS Part III score and selected subtotal scores improved significantly in 12 weeks for both elderly and non-elderly patients. For rotigotine monotherapy in early stage patients, the total UPDRS Part III score improved significantly in non-elderly patients, but not in elderly patients. This phenomenon is probably due to a lack of power associated with the small sample size.

The incidences of total AEs and some remarkable AEs were lower in elderly patients than non-elderly patients for both combination therapy and monotherapy. A noteworthy decrease of the UPDRS score for postural instability was seen in the total patient population and non-elderly patients with combination therapy (Table S3A). There were also differences in improvement for each UPDRS item score between age groups in the combination therapy; items related to motion (falling, freezing when walking, arising from chair) improved in non-elderly patients, and those related to ADLs (depression, hygiene, turning in bed and adjusting bed clothes, gait) improved in elderly patients. Generally, out of the PD motor symptoms, tremor and rigidity can be observed visually and the change in scores is noticeable, whereas it is said to be the opposite for akinesia and postural instability. Postural instability becomes clear after HY stage 3, and presents as a degradation in posture (standing up, tumbling down, pulsion, etc.) [19–21], which is associated with impaired ADL. Further, it is generally accepted that postural instability and ADLs are hard to improve by treatment with drugs. Gait disturbance also interferes with ADL. Somnolence and orthostatic hypotension are major AEs that affect ADL. Orthostatic hypotension occurred less frequently in elderly patients compared to non-elderly patients for combination therapy in advanced stage patients, and did not occur in either age group with monotherapy in early stage patients. It is reported that dopamine receptors (D1–D5) are specifically involved in the regulation of blood pressure [22]. In addition, rotigotine is reported not to influence cardiovascular autonomic responses in de novo PD patients [23]. Consequently, the major reason for the improvement of postural instability and ADLs in patients treated with rotigotine in this study could be due to lower occurrence of these AEs and the pharmacological property of rotigotine that includes a well-balanced response to all dopamine receptors. Moreover, somnolence is also reported to accelerate postural instability [19]. Many drugs lead to somnolence and orthostatic hypotension, which are quite frequent AEs, especially for dopamine agonists [24]; therefore, taking these AEs into account is important when trying to prevent a decline in ADL. However, recent reports described that somnolence is less frequent for rotigotine [25, 26], and that quality of sleep [11, 26–28] and motor functions in the early morning [27] are improved. In this study, gait disturbance improved both in elderly and non-elderly patients, and some UPDRS scores related to ADLs improved more in elderly patients than in non-elderly patients. These results may be related to the lower incidence of somnolence in elderly patients with advanced PD than in non-elderly patients.

Some UPDRS scores also improved in the patients treated with placebo. There are several reports about placebo effect in PD patients [29, 30]. In this study, the items showing a placebo effect were different by age group and treatment type (monotherapy or combination therapy, related to the stage of PD progression) (Fig. 2). Such differences are likely to correspond to observations in clinical practice, and are related to the effect of rotigotine treatment compared to placebo in this study.

The change in OFF-time was small and not significant in elderly patients, whereas significant change was shown among non-elderly patients. It is possible that drug reactivity is greater in non-elderly patients, and the amount of activity is lower in elderly patients, so OFF-time is less noticeable in elderly patients. The tendency not to complain about psychological symptoms compared to motor symptoms may be a reason there was a small change in UPDRS Part I score in patients.

In this study, administration of rotigotine led to a significant improvement in some UPDRS scores, mainly in combination therapy on advanced stage patients. The notable impact of the combination of levodopa and rotigotine may possibly be a synergistic effect like the combination of levodopa and pramipexole, reported previously [31].

In terms of safety, the incidence of AEs tended to be lower in elderly than in non-elderly patients, with both combination therapy and monotherapy. This is despite dopamine agonists being described as inferior to levodopa when treating elderly patients [2]. It was also previously mentioned that age is a risk factor for an AE [32]. Possible reasons for such unexpected results might be the relatively constant blood concentration of rotigotine, and less tendency to complain about symptoms among elderly patients compared to non-elderly patients. Moreover, the lower incidence of digestive symptoms, vomiting and nausea, in elderly patients may be due to the difference in drug reactivity; higher in non-elderly patients than elderly patients. The lower incidence of total and some remarkable AEs in elderly compared to non-elderly patients might not be consistent with impressions from clinical practice either. It is probably due to patients with severe complications being excluded from the trials used in this study. That is, if patients do not have such complications, the total safety results in elderly are not worse compared to non-elderly patients. Lower incidence of some AEs in elderly patients prescribed rotigotine was also reported in a previous study based on randomized clinical trials with both cut-off ages of 65- and 75-years-old [13]. In terms of visual hallucination and hallucination, cumulative incidence rates tended to be higher in elderly patients than in non-elderly patients. The incidence of hallucination for monotherapy was higher than for combination therapy in elderly patients. Clinicians should, therefore, be aware of this when treating elderly patients with rotigotine. In total, the incidence of AEs was lower in elderly patients compared to non-elderly patients.

In the clinical trials used in this study, the dosage of drugs (2–16 mg/24 h) was reduced to a level that could be maintained if it was decided that the patient could not take any more. One reason for this decision was the occurrence of AEs, so we examined the average dosage of drugs when certain AEs (application site reactions, visual hallucination and hallucination, and somnolence) occurred. A large difference in dosage was not found between elderly and non-elderly patients with these AEs (Table 3). Moreover, the average maintenance dosage of rotigotine was approximately the same between elderly and non-elderly patients for combination therapy and monotherapy (Table S2). Consequently, an improvement in symptoms could be expected in elderly patients as well as non-elderly patients.

**Table 3:** Dosage in application site reactions, visual hallucination and hallucination, and somnolence

Trial	Adverse event	Elderly/non-elderly	Group	*n*	Mean dosage^b^ (mg/24 h)	SD
Combination therapy with levodopa	Application site reactions^a^	Elderly	Rotigotine	45	10.1	4.5
			Placebo	16	8.3	5.5
		Non-elderly	Rotigotine	129	8.6	5.2
			Placebo	28	10.2	4.5
	Visual hallucination/hallucination	Elderly	Rotigotine	14	10.3	3.2
			Placebo	4	14.0	2.3
		Non-elderly	Rotigotine	16	10.6	5.5
			Placebo	4	7.5	6.0
	Somnolence	Elderly	Rotigotine	6	8.7	4.5
			Placebo	1	4.0	–
		Non-elderly	Rotigotine	17	7.4	4.8
			Placebo	2	3.0	1.4
Monotherapy	Application site reactions^a^	Elderly	Rotigotine	12	8.7	4.8
			Placebo	6	7.0	4.1
		Non-elderly	Rotigotine	32	8.4	4.9
			Placebo	14	9.1	4.4
	Visual hallucination/hallucination	Elderly	Rotigotine	6	7.7	2.7
			Placebo	–	–	–
		Non-elderly	Rotigotine	2	11.0	1.4
			Placebo	–	–	–
	Somnolence	Elderly	Rotigotine	6	8.7	5.9
			Placebo	1	2.0	–
		Non-elderly	Rotigotine	7	5.7	3.7
			Placebo	3	10.7	5.0

^a^Including application site itching, application site erythema, application site reaction, application site irritation, application site vesicles, application site hypersensitivity, application site dermatitis, application site exfoliation, application site edema, and application site discoloration

^b^The dosage of placebo is defined as a patch of the same size as the rotigotine transdermal patch

There are several limitations in this study. Post hoc analysis was performed by dividing the patients into sub-groups after the clinical trials had concluded; therefore, the group sample size decreased, especially for the monotherapy group. Regarding safety, since the trials used in this study were conducted only for 12 or 16 weeks, the safety of long-term administration is unknown. Finally, the patients in all of the trials were aged 30–79 years; that is, there were no data for patients aged ≥ 80 years.

In conclusion, in this post hoc analysis, elderly patients showed improvement in motor symptoms similar to non-elderly patients, and a tendency to lower the frequency of AEs when treated with a combination therapy of rotigotine and levodopa for advanced PD. Additionally, there was no major difference in the maintenance dosage of rotigotine between elderly and non-elderly patients. The results suggest that the rotigotine transdermal patch has good tolerability and can be used for elderly PD patients with the expectation of an improvement in ADL.

## Electronic supplementary material

Below is the link to the electronic supplementary material.

Supplementary material 1 (PDF 379 kb)
