# Supplementary material for: Efficacy and safety of rotigotine in elderly patients with Parkinson’s disease in comparison with the non-elderly: a post hoc analysis of randomized, double-blind, placebo-controlled trials
Source: J Neurol. 2017 Nov 21;265(2):253–65. doi: 10.1007/s00415-017-8671-0 (PMC5808069; doi:10.1007/s00415-017-8671-0)
Supplement: Supplementary file 1 — Supplementary material 1 (PDF 379 kb) [file 415_2017_8671_MOESM1_ESM.pdf]

Supplementary Material

Journal of Neurology

Efficacy and safety of rotigotine in elderly compared to non-elderly Parkinson’s disease patients: a post-hoc analysis of randomized, double-blind, placebo-controlled trials.

Masahiro Nomoto, Hirotaka Iwaki, Hiroyuki Kondo, Masaya Sakurai

Corresponding author

Masahiro Nomoto

Department of Neurology and Clinical Pharmacology, Ehime University Hospital, Shitsukawa, Ehime, Japan

E-mail: nomoto@m.ehime-u.ac.jp

Table S1. Disposition of patients in each trial.

Trial	SS			FAS		
	Rotigotine	Placebo	Total	Rotigotine	Placebo	Total
Combination therapy with levodopa						
NCT01628848	87	87	174	86	86	172
NCT01628926	168	85	253	164	84	248
total	255	172	427	250	170	420
Monotherapy						
NCT01628965	90	90	180	88	88	176

SS, safety set: patients in the randomized sample who underwent treatment at least once with rotigotine or placebo except those who violated good clinical practice.
FAS, full analysis set: among safety set patients, those who had at least one post-baseline evaluation for efficacy measures and did not violate inclusion/exclusion criteria.

Table S2. Summary of rotigotine dosage in this study.

Trial	Elderly /Non-elderly	Group	Statistic	Maintenance dosage* (mg/24h)	Maximum dosage* (mg/24h)	Mean dosage* (mg/24h)
Combination therapy with levodopa	Elderly	Rotigotine	n	82	86	86
			Mean	13.4	13.6	9.9
			SD	3.8	3.6	2.5
			Minimum	2.0	2.0	2.0
			Median	16.0	16.0	10.9
			Maximum	16.0	16.0	12.4
	Non-elderly	Rotigotine	n	152	164	164
			Mean	12.5	12.7	9.3
			SD	3.9	3.8	2.8
			Minimum	4.0	2.0	2.0
			Median	14.0	14.0	9.8
			Maximum	16.0	16.0	12.4
Monotherapy	Elderly	Rotigotine	n	26	28	28
			Mean	13.1	13.1	9.1
			SD	3.9	3.8	2.4
			Minimum	6.0	4.0	3.1
			Median	16.0	16.0	10.5
			Maximum	16.0	16.0	11.3
	Non-elderly	Rotigotine	n	56	60	60
			Mean	12.7	12.7	8.9
			SD	4.0	4.2	2.6
			Minimum	4.0	2.0	0.0
			Median	16.0	16.0	10.3
			Maximum	16.0	16.0	11.3

SD, standard deviation. *The dosage of placebo is defined as a patch of the same size as the rotigotine transdermal patch.

Table S3. Change in UPDRS scores related to (A) motor symptoms and (B) gait disturbance, from baseline to the end of maintenance period.

(A)

PD motor symptom	Trial	Elderly /Non-elderly	Group	n	Change mean	Treatment comparison (Rotigotine-Placebo)[a) Point estimation	Treatment comparison (Rotigotine-Placebo)[a) 95% confidence interval	Treatment comparison (Rotigotine-Placebo)[a) p value
Tremor[b)]	Combination therapy with levodopa	Elderly	Rotigotine	86	-2.45	-1.63	-2.65, -0.61	0.0020
			Placebo	68	-0.82			
		Non-elderly	Rotigotine	164	-2.10	-1.19	-1.86, -0.52	0.0006
			Placebo	102	-0.91			
		Total	Rotigotine	250	-2.22	-1.34	-1.91, -0.78	< 0.0001
			Placebo	170	-0.88			
	Monotherapy	Elderly	Rotigotine	28	-1.68	-0.74	-1.76, 0.27	0.1486
			Placebo	31	-0.94			
		Non-elderly	Rotigotine	60	-1.93	-1.32	-2.12, -0.51	0.0015
			Placebo	57	-0.61			
		Total	Rotigotine	88	-1.85	-1.13	-1.75, -0.50	0.0005
			Placebo	88	-0.73			
Rigidity[c)]	Combination therapy with levodopa	Elderly	Rotigotine	86	-2.13	-0.7	-1.47, 0.07	0.0735
			Placebo	68	-1.43			
		Non-elderly	Rotigotine	164	-2.68	-1.38	-1.98, -0.78	< 0.0001
			Placebo	102	-1.30			
		Total	Rotigotine	250	-2.49	-1.14	-1.61, -0.67	< 0.0001
			Placebo	170	-1.35			
	Monotherapy	Elderly	Rotigotine	28	-1.86	-0.6	-1.82, 0.63	0.3312
			Placebo	31	-1.26			
		Non-elderly	Rotigotine	60	-1.43	-0.7	-1.52, 0.13	0.0979
			Placebo	57	-0.74			
		Total	Rotigotine	88	-1.57	-0.65	-1.33, 0.03	0.0617
			Placebo	88	-0.92			
Akinesia[d)]	Combination therapy with levodopa	Elderly	Rotigotine	86	-4.79	-2.14	-3.86, -0.43	0.0147
			Placebo	68	-2.65			_
		Non-elderly	Rotigotine	164	-6.13	-3.53	-4.74, -2.32	<0.0001
			Placebo	102	-2.61			
		Total	Rotigotine	250	-5.67	-3.05	-4.04, -2.06	<0.0001
			Placebo	170	-2.62			_
	Monotherapy	Elderly	Rotigotine	28	-3.86	-0.99	-3.41, 1.44	0.4190
			Placebo	31	-2.87			
		Non-elderly	Rotigotine	60	-3.23	-2.01	-3.33, -0.68	0.0033
			Placebo	57	-1.23			
		Total	Rotigotine	88	-3.43	-1.63	-2.82, -0.43	0.0078
			Placebo	88	-1.81			
Postural instability[e)]	Combination therapy with levodopa	Elderly	Rotigotine	86	-0.72	-0.41	-0.94, 0.12	0.1286
			Placebo	68	-0.31			
		Non-elderly	Rotigotine	164	-0.98	-0.72	-1.07, -0.38	< 0.0001
			Placebo	102	-0.25			
		Total	Rotigotine	250	-0.89	-0.61	-0.90, -0.32	< 0.0001
			Placebo	170	-0.28			
	Monotherapy	Elderly	Rotigotine	28	-0.71	-0.29	-1.16, 0.57	0.4986
			Placebo	31	-0.42			
		Non-elderly	Rotigotine	60	-0.40	-0.44	-0.91, 0.04	0.0701
			Placebo	57	0.04			
		Total	Rotigotine	88	-0.50	-0.38	-0.80, 0.05	0.0828
			Placebo	88	-0.13			

a) Equal variances assumed; b)Total score of UPDRS items 16 (tremor), 20 (tremor at rest), and 21 (action or postural tremor of hands); c) Score of UPDRS item 22 (rigidity); d) Total score of UPDRS items 8 (handwriting), 10 (dressing), 11 (hygiene), 12 (turning in bed and adjusting bed clothes), 18 (speech), 19 (facial expression), 23 (finger taps), 24 (hand movements), 25 (rapid alternating movements of hands), 26 (leg agility), and 31 (body bradykinesia and hypokinesia) ; e) Total score of UPDRS items 27 (arising from chair), 28 (posture), and 30 (postural stability).

(B)

Trial	Elderly /Non-elderly	Group	n	Change mean	Treatment comparison (Rotigotine-Placebo)[a)] Point estimation	95% confidence interval	p value
Combination therapy with levodopa	Elderly	Rotigotine	86	-1.07	-0.81	-1.36, -0.25	0.0050
		Placebo	68	-0.26			
	Non-elderly	Rotigotine	164	-1.05	-0.68	-1.07, -0.29	0.0007
		Placebo	102	-0.37			
	Total	Rotigotine	250	-1.06	-0.73	-1.05, -0.41	< 0.0001
		Placebo	170	-0.33			
Monotherapy	Elderly	Rotigotine	28	-0.54	-0.02	-0.82, 0.78	0.9612
		Placebo	31	-0.52			
	Non-elderly	Rotigotine	60	-0.63	-0.58	-1.10, -0.07	0.0273
		Placebo	57	-0.05			
	Total	Rotigotine	88	-0.60	-0.39	-0.82, 0.05	0.0790
		Placebo	88	-0.22			

Total score of UPDRS items 13 (falling), 14 (freezing when walking), 15 (walking), and 29 (gait); a) Equal variances assumed.

Table S4. Change in each UPDRS item score from baseline to the end of maintenance period.

UPDRS item	Trial	Elderly /Non-elderly	Group	n	Change mean	Treatment comparison (Rotigotine - Placebo)[a)] Point estimation	95% confidence interval	p value
3: Depression	Combination therapy with levodopa	Elderly	Rotigotine	86	-0.16	-0.19	-0.36, -0.02	0.0286
			Placebo	68	0.03			
		Non-elderly	Rotigotine	164	-0.15	-0.10	-0.21, 0.01	0.0656
			Placebo	102	-0.05			
		Total	Rotigotine	250	-0.16	-0.14	-0.23, -0.04	0.0039
			Placebo	170	-0.02			
	Monotherapy	Elderly	Rotigotine	28	-0.21	-0.15	-0.37, 0.08	0.1878
			Placebo	31	-0.06			
		Non-elderly	Rotigotine	60	-0.08	-0.14	-0.31, 0.04	0.1350
			Placebo	57	0.05			
		Total	Rotigotine	88	-0.13	-0.14	-0.28, 0.00	0.0565
			Placebo	88	0.01			
4: Motivation /Initiative	Combination therapy with levodopa	Elderly	Rotigotine	86	-0.21	-0.24	-0.44, -0.04	0.0207
			Placebo	68	0.03			
		Non-elderly	Rotigotine	164	-0.22	-0.20	-0.34, -0.06	0.0048
			Placebo	102	-0.02			
		Total	Rotigotine	250	-0.22	-0.22	-0.33, -0.10	0.0002
			Placebo	170	0.00			
	Monotherapy	Elderly	Rotigotine	28	-0.25	-0.15	-0.46, 0.16	0.3248
			Placebo	31	-0.10			
		Non-elderly	Rotigotine	60	-0.07	-0.03	-0.17, 0.10	0.6445
			Placebo	57	-0.04			
		Total	Rotigotine	88	-0.13	-0.07	-0.20, 0.07	0.3237
			Placebo	88	-0.06			
10: Dressing	Combination therapy with levodopa	Elderly	Rotigotine	86	-0.26	-0.17	-0.35, 0.02	0.0790
			Placebo	68	-0.09			
		Non-elderly	Rotigotine	164	-0.21	-0.10	-0.23, 0.03	0.1234
			Placebo	102	-0.11			
		Total	Rotigotine	250	-0.22	-0.12	-0.23, -0.02	0.0208
			Placebo	170	-0.10			
	Monotherapy	Elderly	Rotigotine	28	-0.04	0.19	-0.07, 0.45	0.1521
			Placebo	31	-0.23			
		Non-elderly	Rotigotine	60	-0.13	-0.10	-0.25, 0.05	0.1923
			Placebo	57	-0.04			
		Total	Rotigotine	88	-0.10	0.00	-0.13, 0.13	1.0000
			Placebo	88	-0.10			

(Continued)

UPDRS item	Trial	Elderly /Non-elderly	Group	n	Change mean	Treatment comparison (Rotigotine - Placebo)[a)] Point estimation	95% confidence interval	p value
11: Hygiene	Combination therapy with levodopa	Elderly	Rotigotine	86	-0.19	-0.19	-0.36, -0.02	0.0327
			Placebo	68	0.00			
		Non-elderly	Rotigotine	164	-0.21	-0.12	-0.25, 0.02	0.0905
			Placebo	102	-0.10			
		Total	Rotigotine	250	-0.20	-0.15	-0.25, -0.04	0.0067
			Placebo	170	-0.06			
	Monotherapy	Elderly	Rotigotine	28	-0.07	0.06	-0.30, 0.42	0.7509
			Placebo	31	-0.13			
		Non-elderly	Rotigotine	60	-0.05	-0.09	-0.22, 0.05	0.2020
			Placebo	57	0.04			
		Total	Rotigotine	88	-0.06	-0.03	-0.18, 0.11	0.6485
			Placebo	88	-0.02			
12: Turning in bed and adjusting bed clothes	Combination therapy with levodopa	Elderly	Rotigotine	86	-0.28	-0.23	-0.44, -0.03	0.0250
			Placebo	68	-0.04			
		Non-elderly	Rotigotine	164	-0.24	-0.12	-0.24, 0.00	0.0546
			Placebo	102	-0.12			
		Total	Rotigotine	250	-0.25	-0.16	-0.27, -0.06	0.0029
			Placebo	170	-0.09			
	Monotherapy	Elderly	Rotigotine	28	-0.11	-0.01	-0.33, 0.31	0.9485
			Placebo	31	-0.10			
		Non-elderly	Rotigotine	60	-0.15	-0.06	-0.23, 0.11	0.4645
			Placebo	57	-0.09			
		Total	Rotigotine	88	-0.14	-0.05	-0.20, 0.11	0.5568
			Placebo	88	-0.09			
13: Falling (Unrelated to freezing)	Combination therapy with levodopa	Elderly	Rotigotine	86	-0.19	-0.14	-0.33, 0.05	0.1439
			Placebo	68	-0.04			
		Non-elderly	Rotigotine	164	-0.14	-0.10	-0.20, -0.00	0.0446
			Placebo	102	-0.04			
		Total	Rotigotine	250	-0.16	-0.11	-0.21, -0.02	0.0162
			Placebo	170	-0.04			
	Monotherapy	Elderly	Rotigotine	28	0.07	0.14	-0.16, 0.43	0.3537
			Placebo	31	-0.06			
		Non-elderly	Rotigotine	60	-0.07	0.02	-0.11, 0.15	0.7474
			Placebo	57	-0.09			
		Total	Rotigotine	88	-0.02	0.06	-0.07, 0.19	0.3836
			Placebo	88	-0.08			
14: Freezing when walking	Combination therapy with levodopa	Elderly	Rotigotine	86	-0.24	-0.14	-0.35, 0.07	0.1890
			Placebo	68	-0.10			
		Non-elderly	Rotigotine	164	-0.24	-0.23	-0.39, -0.08	0.0036
			Placebo	102	-0.01			
		Total	Rotigotine	250	-0.24	-0.20	-0.32, -0.07	0.0021
			Placebo	170	-0.05			
	Monotherapy	Elderly	Rotigotine	28	-0.18	-0.15	-0.33, 0.04	0.1167
			Placebo	31	-0.03			
		Non-elderly	Rotigotine	60	-0.17	-0.24	-0.46, -0.01	0.0408
			Placebo	57	0.07			
		Total	Rotigotine	88	-0.17	-0.20	-0.37, -0.04	0.0134
			Placebo	88	0.03			

(Continued)

UPDRS item	Trial	Elderly /Non-elderly	Group	n	Change mean	Treatment comparison (Rotigotine - Placebo)[a)] Point estimation	95% confidence interval	p value
27: Arising from Chair	Combination therapy with levodopa	Elderly	Rotigotine	86	-0.16	-0.12	-0.38, 0.14	0.3653
			Placebo	68	-0.04			
		Non-elderly	Rotigotine	164	-0.30	-0.26	-0.42, -0.10	0.0016
			Placebo	102	-0.04			
		Total	Rotigotine	250	-0.25	-0.21	-0.35, -0.07	0.0029
			Placebo	170	-0.04			
	Monotherapy	Elderly	Rotigotine	28	-0.18	-0.02	-0.34, 0.31	0.9160
			Placebo	31	-0.16			
		Non-elderly	Rotigotine	60	-0.10	-0.15	-0.35, 0.05	0.1332
			Placebo	57	0.05			
		Total	Rotigotine	88	-0.13	-0.10	-0.27, 0.07	0.2399
			Placebo	88	-0.02			
29: Gait	Combination therapy with levodopa	Elderly	Rotigotine	86	-0.36	-0.30	-0.50, -0.10	0.0035
			Placebo	68	-0.06			
		Non-elderly	Rotigotine	164	-0.32	-0.07	-0.22, 0.08	0.3732
			Placebo	102	-0.25			
		Total	Rotigotine	250	-0.34	-0.16	-0.28, -0.04	0.0095
			Placebo	170	-0.18			
	Monotherapy	Elderly	Rotigotine	28	-0.21	-0.02	-0.29, 0.25	0.8795
			Placebo	31	-0.19			
		Non-elderly	Rotigotine	60	-0.20	-0.22	-0.41, -0.03	0.0244
			Placebo	57	0.02			
		Total	Rotigotine	88	-0.20	-0.15	-0.30, 0.01	0.0611
			Placebo	88	-0.06			
30: Postural Stability	Combination therapy with levodopa	Elderly	Rotigotine	86	-0.33	-0.13	-0.37, 0.10	0.2595
			Placebo	68	-0.19			
		Non-elderly	Rotigotine	164	-0.39	-0.18	-0.34, -0.03	0.0216
			Placebo	102	-0.21			
		Total	Rotigotine	250	-0.37	-0.17	-0.30, -0.04	0.0120
			Placebo	170	-0.20			
	Monotherapy	Elderly	Rotigotine	28	-0.21	-0.12	-0.52, 0.28	0.5585
			Placebo	31	-0.10			
		Non-elderly	Rotigotine	60	-0.18	-0.11	-0.33, 0.10	0.3059
			Placebo	57	-0.07			
		Total	Rotigotine	88	-0.19	-0.11	-0.31, 0.08	0.2498
			Placebo	88	-0.08			

a) Equal variances assumed

Table S5. Change in OFF time from baseline to the end of maintenance period.

					Treatment comparison (Rotigotine - Placebo)[a)]		
Trial	Elderly /Non-elderly	Group	n	Change mean	Point estimation	95% confidence interval	p value
Combination therapy with levodopa	Elderly	Rotigotine	49	-1.03	-0.24	-1.43, 0.96	0.6951
		Placebo	40	-0.79			
	Non-elderly	Rotigotine	116	-1.99	-1.49	-2.32, -0.65	0.0005
		Placebo	73	-0.50			
	Total	Rotigotine	165	-1.71	-1.10	-1.78, -0.42	0.0017
		Placebo	113	-0.61			

a) Equal variances assumed.

Table S6. Change in total UPDRS Part I score from baseline to the end of maintenance period.

					Treatment comparison (Rotigotine - Placebo)[a)]		
Trial	Elderly/Non-elderly	Group	n	Change mean	Point estimation	95% confidence interval	p value
Combination therapy with levodopa	Elderly	Rotigotine	86	-0.24	-0.32	-0.72, 0.08	0.1170
		Placebo	68	0.07			
	Non-elderly	Rotigotine	164	-0.31	-0.25	-0.52, 0.02	0.0656
		Placebo	102	-0.06			
	Total	Rotigotine	250	-0.29	-0.28	-0.51, -0.06	0.0133
		Placebo	170	-0.01			
Monotherapy	Elderly	Rotigotine	28	-0.39	-0.23	-0.79, 0.33	0.4086
		Placebo	31	-0.16			
	Non-elderly	Rotigotine	60	-0.18	-0.20	-0.49, 0.09	0.1783
		Placebo	57	0.02			
	Total	Rotigotine	88	-0.25	-0.20	-0.47, 0.06	0.1326
		Placebo	88	-0.05			

a) Equal variances assumed.

Table S7. Change in sum of UPDRS Part II and Part III scores from baseline to the end of maintenance period.

					Treatment comparison (Rotigotine - Placebo)[a)]		
Trial	Elderly /Non-elderly	Group	n	Change mean	Point estimation	95% confidence interval	p value
Monotherapy	Elderly	Rotigotine	28	-9.25	-2.77	-7.89, 2.36	0.2847
		Placebo	31	-6.48			
	Non-elderly	Rotigotine	60	-8.02	-5.24	-8.33, -2.16	0.0010
		Placebo	57	-2.77			
	Total	Rotigotine	88	-8.41	-4.33	-7.00, -1.66	0.0016
		Placebo	88	-4.08			

a) Equal variances assumed.
